# A new manual dispensing system for *in meso* membrane protein crystallization with using a stepping motor-based dispenser

**DOI:** 10.1007/s10969-014-9187-9

**Published:** 2014-07-24

**Authors:** Masakatsu Hato, Toshiaki Hosaka, Hiroaki Tanabe, Tokuji Kitsunai, Shigeyuki Yokoyama

**Affiliations:** 1 RIKEN Systems and Structural Biology Center, 1-7-22 Suehiro-cho, Tsurumi-ku, Yokohama, Kanagawa 230-0045 Japan; 2 Division of Structural and Synthetic Biology, RIKEN Center for Life Science Technologies, 1-7-22 Suehiro-cho, Tsurumi-ku, Yokohama, Kanagawa 230-0045 Japan; 3 Advanced Photonics Technology Development Group, RIKEN Center for Advanced Photonics, 2-1 Hirosawa, Wako, Saitama 351-0198 Japan; 4 RIKEN Structural Biology Laboratory, 1-7-22 Suehiro-cho, Tsurumi-ku, Yokohama, Kanagawa 230-0045 Japan

**Keywords:** *In meso* membrane protein crystallization, Dispenser, Manual dispensing, Cubic phase, Membrane protein, Bacteriorhodopsin

## Abstract

A reliable and easy to use manual dispensing system has been developed for the *in meso* membrane protein crystallization method. The system consists of a stepping motor-based dispenser with a new microsyringe system for dispensing, which allows us to deliver any desired volume of highly viscous lipidic mesophase in the range from ~50 to at least ~200 nl. The average, standard deviation, and coefficient of variation of 20 repeated deliveries of 50 nl cubic phase were comparable to those of a current robotic dispensing. Moreover, the bottom faces of boluses delivered to the glass crystallization plate were reproducibly circular in shape, and their centers were within about 100 μm from the center of the crystallization well. The system was useful for crystallizing membrane and soluble proteins *in meso*.

## Introduction

*In meso* crystallization [1] has proven to be a powerful technique for the crystallization of a variety of membrane proteins, which are difficult to crystallize by other methods [2–4]. The requirements for handling viscous lipidic mesophases, particularly cubic phases, and screening a huge compositional/environmental space to identify optimum crystallization conditions have led to the development of various new tools, such as a microsyringe-based mixing device [5], a 96-well glass crystallization plate [6], and an *in meso* crystallization robot [7]. The robot can typically dispense **~**50 nl of mesophase per well [5]. On the other hand, for small-scale screening in the optimization steps, for example, manual dispensing is often more convenient than robotic dispensing, and a positive-displacement syringe-based mechanical dispenser was implemented for this purpose [8].

However, there are several problems with the operation of the manual dispenser. First, it is difficult to maintain an optimum needle tip-glass plate distance, *L*, during dispensing. It has been well established that accurate and reproducible volume dispensing can only be achieved when *L* is kept at an optimum value [7]. Second, it is challenging to set a desired dispensing volume at will. Finally, it is difficult to dispense a mesophase reproducibly at the center of the well, and the dispensed mesophase boluses often have irregular shapes, which may lead to irreproducible crystallization kinetics.

We now report a newly configured manual dispensing system for the *in meso* method, which largely overcomes the abovementioned difficulties. The system includes a stepping motor-based dispenser and a new microsyringe system for dispensing. The microsyringe system consists of (1) a new removable syringe needle, (2) a 96-hole polyester dual adhesive sheet for constructing a 96-well glass crystallization plate, (3) a 96-hole spacer plate, which serves as a spacer to secure an optimum value of *L*, and as a centering jig for dispensing, and (4) a lipid-protein homogenization device.

## Materials and methods

### Materials

**Fig. 1:**
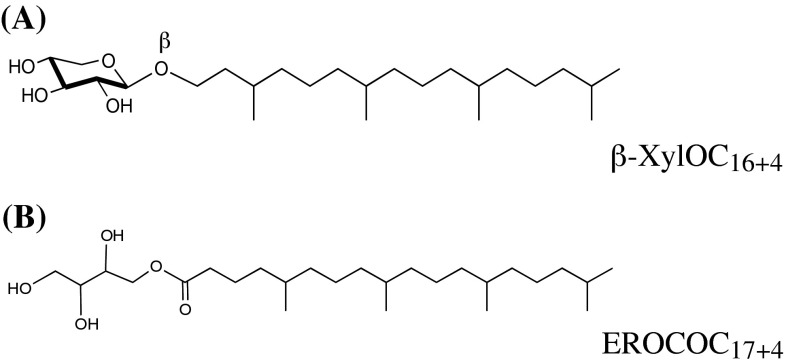
Chemical structures of cubic phase-forming lipids. (**A**) 1-*O*-(3,7,11,15-tetramethylhexadecyl)-β-d-xylopyranoside (β-XylOC_16+4_), (**B**) 1-*O*-(5,9,13,17-tetramethyloctadecanoyl)erythritol (EROCOC_17+4_)

We employed two isoprenoid chained lipid species, 1-*O*-(3,7,11,15-tetramethylhexadecyl)-β-d-xylopyranoside (β-XylOC_16+4_) and 1-*O*-(5,9,13,17-tetramethyloctadecanoyl)erythritol (EROCOC_17+4_), as cubic phase forming lipids. β-XylOC_16+4_ and EROCOC_17+4_ were the same materials described in the previous papers [9, 10, 12], and the chemical structures are shown in Fig. 1. Lysozyme (cat: L-6876) and Bacteriorhodopshin from *Halobacterium salinarum* (cat: B0184) were obtained from Sigma (St. Louis, Mo. USA).

Microsyringes and needles used in this work were obtained from Ito Co. (Fuji, Japan). Glass plates were obtained from Matsunami Glass Ind., Ltd., (Osaka, Japan).

### Homogenization of lipid-water or lipid-protein buffer mixtures

Homogenization of the lipid/water or lipid/protein buffer mixtures was performed at room temperatures (20–23 °C) by using a device consisting of a pair of Ito MS-GAN025 (250 μl) microsyringes with a homebuilt monolithic stainless steel coupler (Fig. 3C). The detailed structure and the homogenization procedures of this device were described in a previous paper [11], where the achievement of molecular homogeneity of lipid/water mixtures was confirmed by a small angle X-ray scattering measurements [10–12]. Though the homogenization procedures were essentially the same as those in the standard *in meso* protocol [5], there was one major difference in the lipid-loading step. The lipids used in this work are viscous waxy material over a temperature range from 0 to at lest ~80 °C [10, 12]. Thus, the lipids (usually 10–30 mg) were loaded into the open end of the microsyringe at room temperatures (without heating) by using a homebuilt stainless steal microspatula followed by an immediate homogenization with water or a protein buffer. In loading solid monoacylglycerols, e.g., monoolein, heating of the lipid at ~40 °C before or after the lipid loading is required [5].

### Estimation of the volume of the delivered cubic phase bolus

The actual volumes of the delivered cubic phase boluses were quantified by processing microscopic images of delivered boluses, which were captured by a BX51 microscope equipped with a C-4040 digital camera (Olympus Co., Tokyo, Japan) [7]. As seen from typical bolus images in Fig. 6B, the bottom faces of delivered boluses were approximately circular in shape after being sandwiched between the two glass plates. We therefore assumed that the delivered boluses are circular truncated cones, so that the bolus volume, *V*, could be estimated from the equation, $V = \frac{1}{3}\left( {S_{1} + \sqrt {S_{1} S_{2} } + S_{2} } \right)d$, where $S_{1}$, $S_{2}$, and *d* denote the upper and lower bottom face areas of the delivered bolus and the well thickness, respectively. The *S* values of each bolus image were quantified by the polygonal area analysis of Image-J (Bioarts Co., Fukuoka, Japan). Each image was calibrated with a NOB1 (pitch 0.01 mm) objective micrometer (Nikon, Co., Tokyo, Japan) to express the *S* value in the SI unit. The *d* value of each well was measured by a VK-8500 confocal microscopy (Kyence, Osaka, Japan) and was found to be 135 ± 2 μm.

A new manual dispensing system for the *in meso* method

### Dispenser

**Fig. 2:**
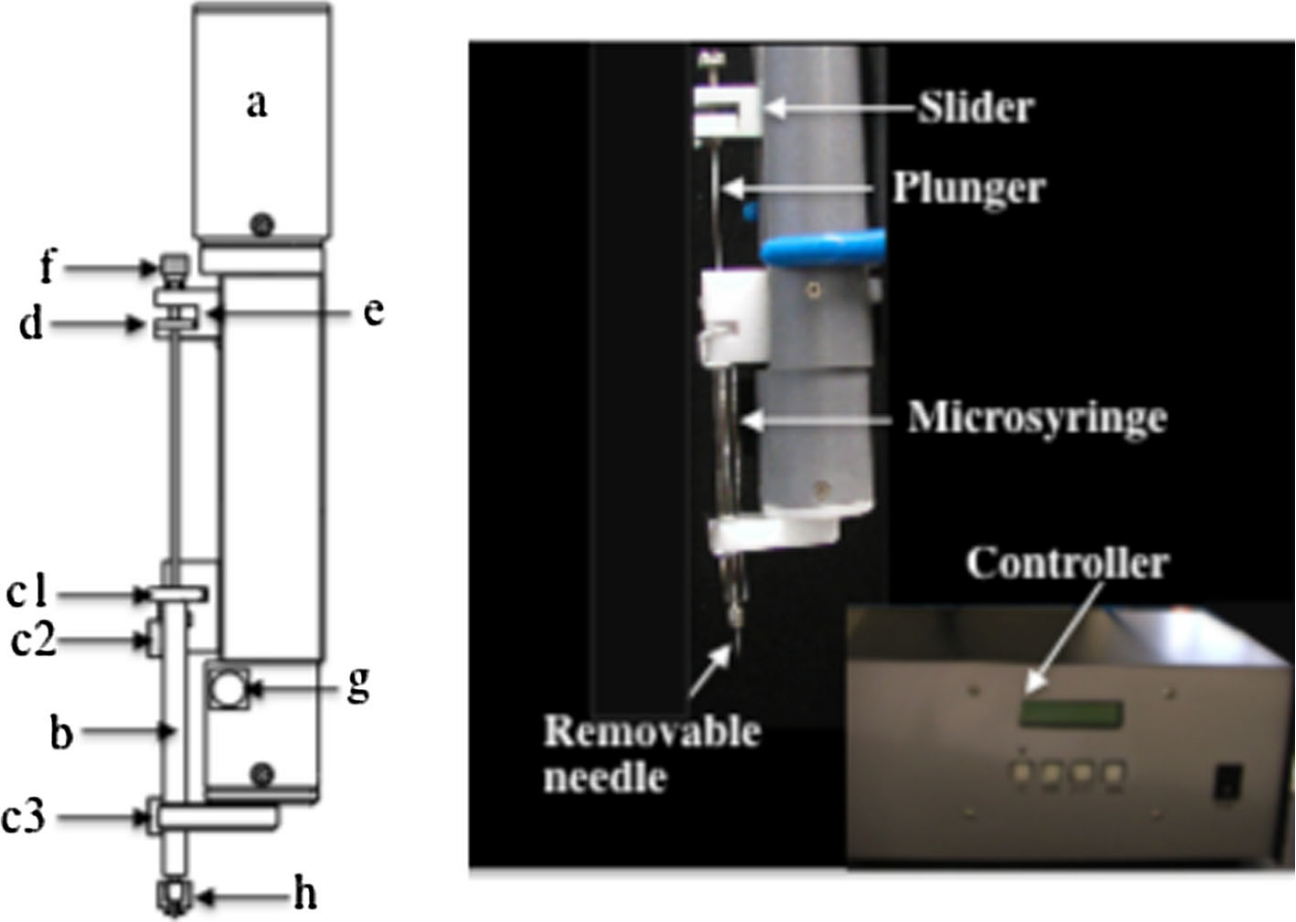
Schematic diagram of the stepping motor-based dispenser

Figure 2 shows a schematic diagram of the stepping motor-based dispenser. As an actuator for dispensing, we employed a KSS model MB0601 5-phase stepping motor (TS3664N17E4) (KSS Co., Ltd. Tokyo, Japan), which moves by 2 μm per pulse (a). The actuator was housed in a 35 mm diameter, cylindrical plastic case. The microsyringe (b), in which the desired mesophase was loaded, was fixed at three positions, c1 **(**the barrel head), c2 and c3 by two magnetic clamps. The plunger head (d) was fixed at a slider (e) by a setscrew (f). The trigger switch (g) is for the actuator. The cubic phase loaded within the microsyringe is expelled onto the glass plate by a motor-driven plunger motion through a removable needle (h**)**. The dispenser can accept any microsyringe type, but we here used Ito gas tight microsyringes, an MS-GFN25 (25 μl), an MS-GFN50 (50 μl), and an MS-GFN100 (100 μl), for which a full stroke is 60 mm (=6.0 × 10^4^ μm). By using the values of 2 μm per pulse and 6.0 × 10^4^ μm for full stroke length, the number of pulses per trigger, *P*, to deliver a desired volume, *v*(nl), can be obtained by the following relation,

1 $$P = \frac{{ 3\times \text{10}^{\text{4}} v}}{{V_{S} }},$$

where *V*_*s*_ (nl) denotes the syringe volume employed for dispensing. For example, with a 100 μl (*V*_*s*_ = 10^5^ nl) syringe, the *P* values to dispense 25, 50, and 100 nl of cubic phase are 8, 15, and 30 pulses/trigger, respectively. With a 50 μl (*V*_*s*_ = 5 × 10^4^ nl) syringe, the corresponding *P* values are 15, 30 and 60 pulses/trigger, respectively.

### The new microsyringe system

#### The removable needle (Fig. 3A)

**Fig. 3:**
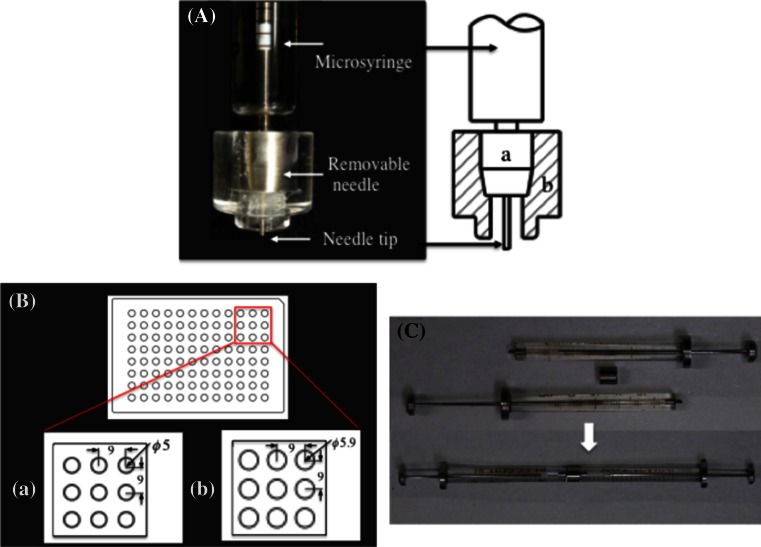
The new microsyringe-based system for dispensing. **A** The new removable needle consisting of an Ito microsyringe needle (*a*) and a fitting ring (*b*). **B** A schematic diagram of the 96-hole polyester dual adhesive sheet (*a*) and the 96-hole spacer plate (*b*). Dimensions are given in millimeters. **C** The lipid-protein homogenization device with the monolithic stainless steel coupler

The new removable needle consists of a commercial Ito 21 or 24 gauge microsyringe needle, with the tip cut at 90° (a), and a homebuilt acrylic fitting ring (b) (Fig. 3A). The fitting ring **(**5.8 mm tip diameter) was designed to fit a hole in the spacer plate [5.9 mm hole diameter, see Fig. 3B-(b)]. The needle length is typically 5 mm (or 2 mm for conservation of materials). The removable needles were used in combination with Ito gas-tight microsyringes, MS-GFN25 (25 μl), MS-GFN50 (50 μl), and MS-GFN100 (100 μl), depending on the desired target volume to be dispensed.

#### The 96-well glass sandwich plate (Fig. 3B-(a))

The 96-well glass crystallization plates used in the present application were made from a perforated 96-hole double-stick polyester sheet (5 mm hole diameter with a standard 9 mm separation and a 1 mm thick Matsunami S100603 glass plate with a footprint of 127.8 × 85.5 mm, which conforms to the Society for Biological Screening (SBS) standard for microplates [5, 7]. As described below, the thickness accuracy of the 96-hole double-stick sheet is critical to ensure the optimum value of *L*, and thus we utilized specifically modified commercial 96-hole double-stick sheets (Kajixx Co., Kawasaki, Japan). The thicknesses of the modified 96-hole double-stick sheet (including the two protecting sheets for the back and front glue layers) and the protective sheet were 235 ± 2 and 50 ± 1 μm, respectively, which we considered satisfactory for the dimensional accuracy required for the present purpose.

#### The 96-hole spacer (Fig. 3B-(b))

**Fig. 4:**
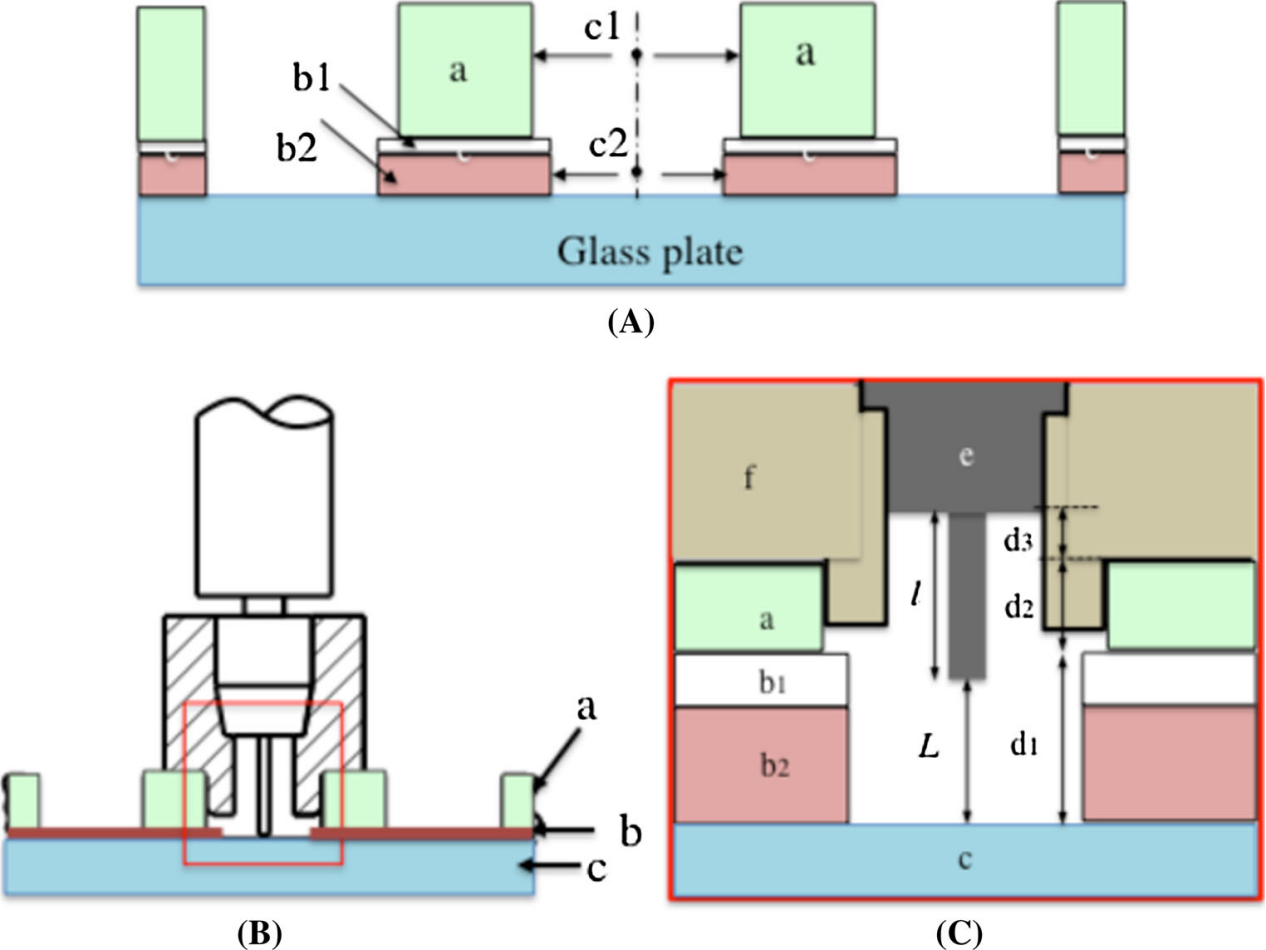
Standard procedure for manual dispensing. **A** A 96-hole spacer plate (*a*) is superimposed on top of a 96-well glass crystallization plate. The front protecting sheet and the 96-hole double-stick sheet are denoted by b1 and b2, respectively. The center of the spacer plate hole and the center of the well are denoted by c1 and c2, respectively. **B** The removable needle is fully inserted into (*a*) the spacer hole. (*b*) The 96-hole double-stick sheet. (*c*) The glass plate. **C** A schematic drawing of the syringe tip (*e*) region, where the needle tip-to-glass plate distance *L* is fixed at a desired value by the relation $L = d_{1} + d_{2} + d_{3} - l$, where $d_{1}$, $d_{2}$, and $d_{3}$ are the thicknesses of the 96-hole double-stick sheet (b1 + b2), the 96-hole spacer plate (*a*), and the fitting ring (*f*), respectively. $l$ is the syringe tip (*e*) length. The figures serve for illustrative purposes only, and are not drawn to scale

The homebuilt 96-hole spacer was made from a 2.95 mm thick acrylic plate, and contains 96 holes. The arrangement of the holes is identical to that of the 96-hole double-stick sheet, except the hole diameter is 5.9 mm, instead of 5 mm for the 96-hole double-stick sheet. Thus, the 96-hole spacer plate serves not only as a spacer to secure an optimum value of *L*, but also as a centering jig for dispensing mesophase boluses (see also Fig. 4).

### Standard procedure for manual dispensing (Fig. 4)

The manual dispensing performance depends on the reproducibility and accuracy of the delivery of the lipid/protein mesophases. As pointed out by Cherezov et al. [7], the reproducibility and accuracy are a complex function of various factors: mesophase composition and volume, needle tip-glass plate distance *L*, needle-tip profile and diameter, and time profile of the delivery cycle. According to our experience, once the tip profile (cut at 90°), and the diameter (21 or 24 gauge) are fixed, the most important factors are the value of *L* and the time profile of the delivery cycle. We systematically examined the effects of these factors on the delivery results, and have established the following standard procedure.

1. A 96-hole spacer plate is superimposed on top of a 96-well glass crystallization plate in such a way that the hole center, c1, is aligned with that of the well center, c2 (Fig. 4A).
2. The removable needle is fully inserted into the desired spacer hole (Fig. 4B). At this stage, the syringe tip position is set at the well’s center, and *L* is fixed at the desired value by the relation $L = d_{1} + d_{2} + d_{3} - l$, where $d_{1}$, $d_{2}$, $d_{3}$, and $l$ are the thicknesses of the 96-hole double-stick sheet, the 96-hole spacer plate, and the fitting ring, and the length of the syringe tip, respectively (Fig. 4C).
3. Trigger a dispenser to deliver a desired mesophase volume and wait for ~1 s.
4. Withdraw the removable needle, move it to the next spacer hole, and insert it.
5. Repeat operations 3 and 4 until all of the intended wells contain dispensed boluses.

## Results and discussion

### Intrinsic accuracy and reproducibility of the dispenser

The performance characteristics of the new method were quantified in two steps.

In the first step, the intrinsic accuracy and reproducibility of the dispenser were evaluated, where the needle tip-glass plate distance, *L*, was controlled with the aid of a manual Z-axis micrometer stage. The dispenser with an Ito MS-GFN25 microsyringe (25 μl) and a 21 gauge removable needle was firmly fixed in a vertical stand, and a 96-well glass crystallization plate was placed on a TSD-601S Z-axis micrometer stage (Sigmakoki Co., Ltd., Tokyo, Japan). On the basis of preliminary experiments, we employed 300, 200, 150, and 125 mm as optimum values of *L* for dispensing 200, 100, 50 and 25 nl cubic phase boluses, respectively. The desired volumes of cubic phase were dispensed consecutively into the wells of a 96-well glass crystallization plate. After the cubic phase was dispensed, 0.8 μl water was overlaid onto each cubic phase and immediately covered with a Matsunami S1100 glass cover slip to compress the water and the cubic phase between the two glass plates. As a cubic phase sample, we used 68 wt% β-XylOC_16+4_, which corresponds to a pure *Pn3m* cubic phase [10].

**Fig. 5:**
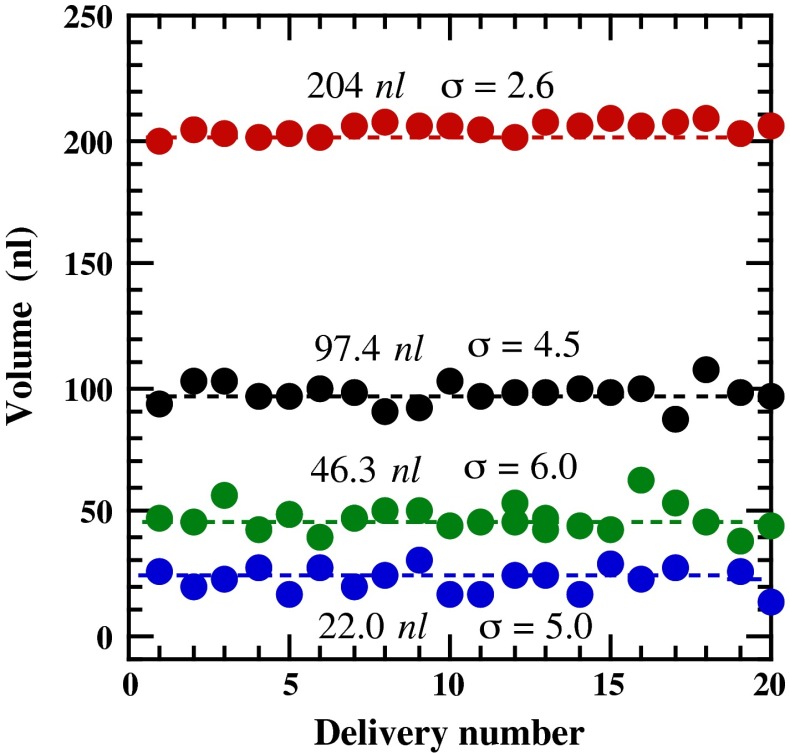
Repetitive measurements of the delivered volumes of a cubic phase, using a 25 μl microsyringe and a 21 gauge removable needle. The delivery conditions were: *red circles* 200 nl (240 pulses/trigger, *L* = 300 μm), *black circles* 100 nl (120 pulses/trigger, *L* = 200 μm), *green circles* 50 nl (60 pulses/trigger, *L* = 150 μm), and *blue circles* 25 nl (30 pulses/trigger, *L* = 125 μm)

Repetitive measurements were performed using 200 nl (red circles), 100 nl (black circles), 50 nl (green circles) and 25 nl (blue circles) as target dispensing volumes, as shown in Fig. 5. The average of the actually dispensed volumes, the standard deviation (σ) and the coefficient of variation (CV) for each target volume were 204 nl (σ = 2.6 nl, CV = 1.3 %), 97.4 (σ = 4.5 nl, CV = 4.6 %), 46.3 (σ = 6.0 nl, CV = 13 %), and 22.5 (σ = 5.0 nl, CV = 22 %), respectively. The results in Fig. 5 indicated that when a target volume was above 100 nl, the actual dispensed volume agreed reasonably well with the target volume (<5 %), whereas deviations from the desired values tended to occur as the target volume decreased, due in part to the complex rheological nature of the cubic phase. Thus, a correction factor may be applied when more accurate dispensing is required (see the next section). The smallest volume that could be dispensed with the present system was about 25 nl. However, the reproducibility was considerably lower than those of volumes ≥50 nl, and extreme perseverance was required to reach the reported reproducibility level. For these reasons, we considered ~50 nl to be the most reasonable lowest volume for the dispenser, to perform manual crystallization trials without great difficulty.

Though we did not systematically examine the maximum dispensable volume, it may be concluded that with this system, a dispensing volume can be set at will over a volume range from ~50 to at least ~200 nl.

### Manual dispensing of 50 nl cubic phase using the new syringe system

**Fig. 6:**
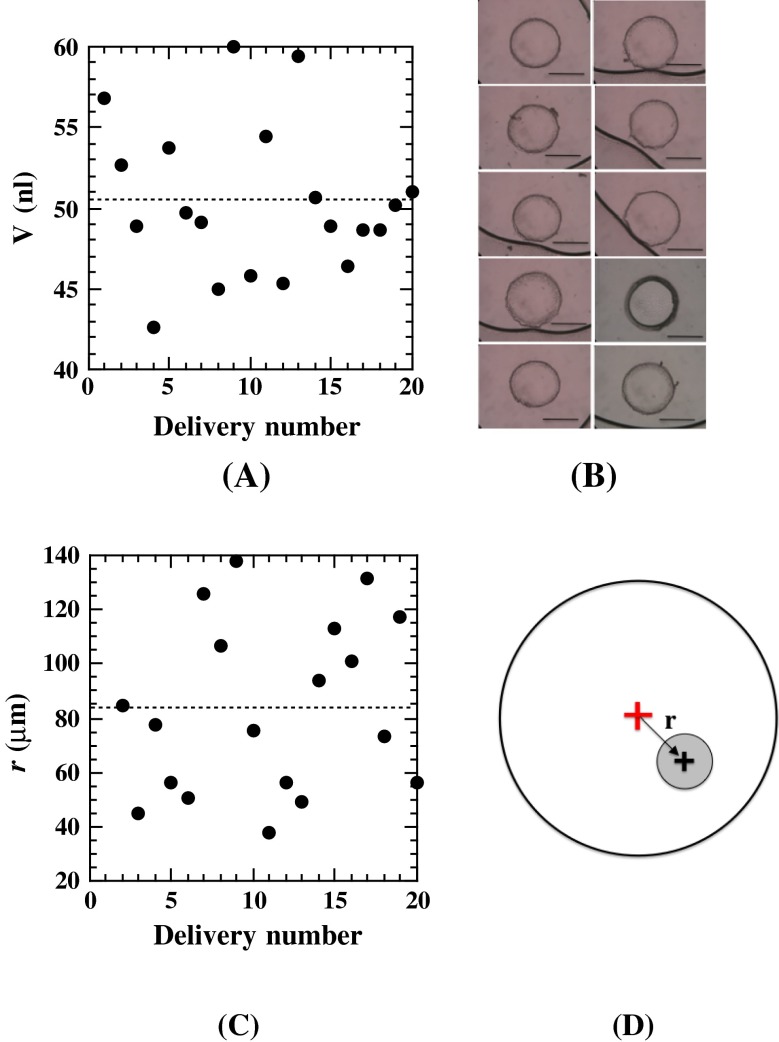
**A** Repetitive measurements of the delivered volumes of a cubic phase with the conditions of *P* = 35 pulses/trigger and *L* = 135 μm. The average actually dispensed volumes, the standard deviation and the coefficient of variation were 50.4, 4.8 nl, and 9.5 %, respectively. **B** Typical images of the delivered cubic phase boluses of 10 consecutive 50 nl dispensed boluses (*bar* 500 μm). **C** Repetitive measurements of the deviation, ***r***, of the delivered bolus center with respect to the well center. **D** The definition of ***r*** for a delivered bolus center (*black cross*) with respect to the well center (*red cross*). The well diameter is 5 mm. The figure is not drawn to scale

In this operation, we employed an Ito MS-GFN50 (50 μl) microsyringe with a 24 gauge removable needle. The value of *L* was set at 135 μm. We first performed 10 repetitive measurements of 50 nl dispensing volumes, using the calculated value of P = 30 pulses/trigger. The average of the actual dispensed volumes was found to be 43 nl, yielding a correction factor of 1.15. We therefore used P = 35 pulses/trigger to dispense 50 nl of mesophase. The results from 20 repetitive dispensings of the β-XylOC_16+4_ cubic phase, using the corrected value of P, are described in Fig. 6 **A**. The average of the actual dispensed volumes, the standard deviation (σ) and the coefficient of variation (CV) were 50.4, 4.8 nl, and 9.5 %, respectively. It is noteworthy that these values were comparable to the robotic 50 nl dispensing [7]. Typical bolus images of 10 consecutive dispensing deliveries, from number 6 to 15 in Fig. 6 **A**, are shown in Fig. 6 **B**, indicating that all of the bottom faces of the boluses were approximately circular in shape.

In Fig. 6 **C**, the deviation, **r**, of each delivered bolus center with respect to the cell center was plotted, where **r** was defined as described in Fig. 6d and evaluated by the image analyses of wells and dispensed boluses. The average value of **r** and the standard deviation were 84 and 32 μm, respectively, with a maximum deviation of ~140 μm. This means that the average deviation of the dispensed boluses is about 3 % (5.6 % at the greatest) of the well radius, 2,500 μm. We considered these results to indicate that the 96-hole plate works well as a centering jig for dispensing the boluses.

### Crystallization trials using the present dispensing system

**Fig. 7:**
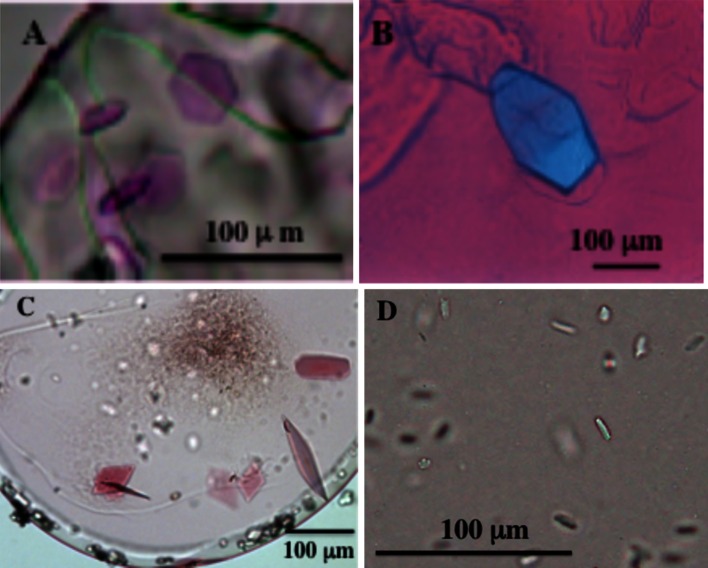
Protein crystals grown by using the new manual dispensing system. Bacteriorhodopsin/β-XylOC_16+4_ (**A**), lysozyme/EROCOC_17+4_ (**B**), proteorhodpsin/EROCOC_17+4_ (**C**), and human hormone receptor/EROCOC_17+4_ (**D**). All crystallizations were performed at 20 °C, except for that of the lysozyme/EROCOC_17+4_ system, which was performed at 4 °C

To further demonstrate the performance of the new manual dispensing system, we performed *in meso* crystallization trials of several membrane proteins, as well as water-soluble lysozyme, using β-XylOC_16+4_ and EROCOC_17+4_ as matrix lipids for crystallization. We followed the standard *in meso* crystallization procedure [5], with 50 nl protein-laden mesophases and 1–0.8 μl crystallization solutions. All crystallizations were performed at 20 °C except for the lysozyme/EROCOC_17+4_ system, which was performed at 4 °C. The protein crystals thus obtained are shown in Fig. 7.

## Concluding remarks

We have described a new manual dispensing system that enables the delivery of highly viscous mesophases used for *in meso* membrane protein crystallizations in a range from ~50 to at least ~200 nanoliter. The accuracy and reproducibility of the delivered mesophase volume achieved by the present dispensing system were shown to be comparable to those of the current robotic dispensing system. Moreover, the bottom faces of the dispensed mesophase boluses were reproducibly circular in shape, and the bolus centers resided within about 100 μm from the center of the well. The system has been used successfully for the *in meso* crystallization of soluble and membrane proteins, and is now in routine use in our laboratory.

Among the tools described in the present report, the lipid-protein homogenization device (Fig. 3C) is now available from Ito Co., Japan. The motor-based dispenser with the new syringe system and 96-well glass crystallization plates are being planned to go on sale in the near future from Ito Co., Japan and Kajixx Co.,/Matsunami Glass Ind., Ltd., Japan, respectively.
